# Utilization of metal or non-metal-based functional materials as efficient composites in cancer therapies

**DOI:** 10.1039/d1ra08335j

**Published:** 2022-02-24

**Authors:** Xiaoxiao He, Shiyue Chen, Xiang Mao

**Affiliations:** State Key Laboratory of Ultrasound in Medicine and Engineering, College of Biomedical Engineering, Chongqing Medical University Chongqing 400016 P. R. China maox@cqmu.edu.cn; Chongqing Key Laboratory of Biomedical Engineering, College of Biomedical Engineering, Chongqing Medical University Chongqing 400016 P. R. China

## Abstract

There has been great progress in cancer treatment through traditional approaches, even though some of them are still trapped in relative complications such as certain side effects and prospective chances of full recovery. As a conventional method, the immunotherapy approach is regarded as an effective approach to cure cancer. It is mainly promoted by immune checkpoint blocking and adoptive cell therapy, which can utilize the human immune system to attack tumor cells and make them necrose completely or stop proliferating cancer cells. Currently however, immunotherapy shows limited success due to the limitation of real applicable cases of targeted tumor environments and immune systems. Considering the urgent need to construct suitable strategies towards cancer therapy, metallic materials can be used as delivery systems for immunotherapeutic agents in the human body. Metallic materials exhibit a high degree of specificity, effectiveness, diagnostic ability, imaging ability and therapeutic effects with different biomolecules or polymers, which is an effective option for cancer treatment. In addition, these modified metallic materials contain immune-modulators, which can activate immune cells to regulate tumor microenvironments and enhance anti-cancer immunity. Additionally, they can be used as adjuvants with immunomodulatory activities, or as carriers for molecular transport to specific targets, which results in the loading of specific ligands to facilitate specific uptake. Here, we provide an overview of the different types of metallic materials used as efficient composites in cancer immunotherapy. We elaborate on the advancements using metallic materials with functional agents as effective composites in synergistic cancer treatment. Some nonmetallic functional composites also appear as a common phenomenon. Ascribed to the design of the composites themselves, the materials' surface structural characteristics are introduced as the drug-loading substrate. The physical and chemical properties of the functional materials emphasize that further research is required to fully characterize their mechanism, showing appropriate relevance for material toxicology and biomedical applications.

## Abstract 2

There has been great progress in cancer treatment through traditional approaches, even though some of them are still trapped in relative complications such as certain side effects and prospective chances of full recovery.

## Graphical abstract

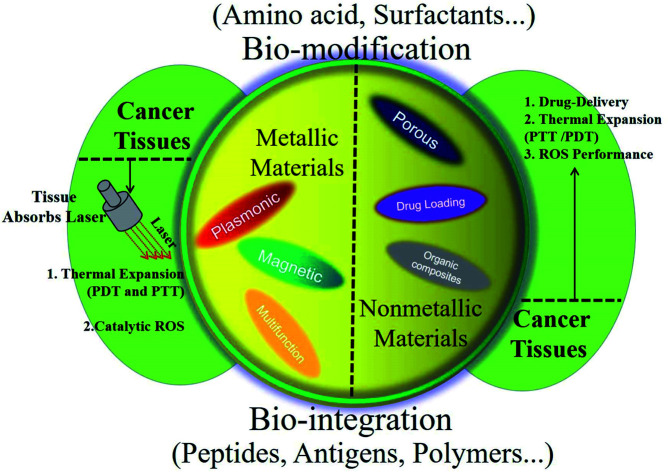

## Introduction

As well demonstrated by decades of research, cancer is commonly considered one of the deadliest diseases worldwide, and is a hot topic of concern in each healthy person's life. Despite a huge number of advances in using functional materials such as organic polymers, metals, and metal-based functional composites to develop new cancer therapies, only limited success has been achieved with current clinical treatment options due to the complexity, diversity, and heterogeneity of tumors.^1,2^ Traditional cancer therapies are mainly based on chemotherapy and radiotherapy. In these forms, the integration of complex diagnostic approaches can be established and utilized completely by combining responsible agents with target tissues. Although the result reflects a strong ability to kill cancer cells, healthy tissues are also adversely affected by serious blood toxicity and tissue dysfunction. Hence, it is necessary to fabricate an effective path in basic cancer therapies. Currently, cancer immunotherapy has aroused widespread attention in the field of tumor therapy. In immunotherapy, the cancer targets are specifically recognized by strengthening the host's immune system. In this system, the major histocompatibility complex and dendritic cells are activated once tumor cells release a series of tumor-specific or associated antigens through overexpression. After this, tumor-derived antigens can be collected and migrated to lymph nodes, and then degraded into peptides as much as possible.^3^ Meanwhile, peptides could express on the surface of dendritic cells (DC) through major histocompatibility complex class II (MHCII) molecules, so they can be recognized by T cells in lymph nodes, thus activating the specific immune response to the tumor site.^4^ As one real immunotherapy application, although cytotoxic T lymphocytes and helper T cells are targeted effective influences, the single action of antigen-presenting cells against antigen uptake is often not enough to induce the immune response required by the immune system.^5,6^ Therefore, in order to match urgent requirements, the development of cancer immunotherapy is mainly focused on the development of an immune regulation system for enhancing the cancer immune response. Additionally, functional metallic materials have been widely used as an effective cancer treatment strategy, it could convey high quality in real diagnosis and cure owing to physical and chemical functions, the relative properties could be adjusted by controlling the composited elements and outer morphology as much as possible.

Herein, one possibility of introducing and utilizing metal-based materials as effective composites in cancer immunotherapies is reported. Metallic materials include different types, which are treated as hopeful consequences to address the limitations of existing cancer immunotherapies.^7,8^ Similarly, the biocompatibility of metallic materials has been extensively studied in the field of drug delivery. It is comprehensively illustrated in light of their ability to efficiently deliver drugs to target sites, protect drugs from endogenous enzymes, and remain in circulation for a long time. It not only conveys acceptable properties but also nonmetallic materials can illustrate the same influences in applied cancer immunotherapies. Also, the liposomes, dendrimers, micelles, polymer particles, and carbon nanotubes can deliver therapeutic drugs to targeting sites.^9,10^ Among them, the utilized nonmetallic materials could allow cancer-specific drug delivery by inherent passive targeting and adopted active targeting strategies in improving pharmaceutics of the loaded drugs.^11^ In addition, the drug could be loaded with metallic materials, the capacity of whole composites should bring more biocompatibility and improve the safety in order to prolong its circulation time and enhance therapeutic outcomes.^12,13^ Therefore, it gained a growing interest in biological and biomedical appropriation toward cancer immunotherapies. The current strategies for immunomodulation have addressed the efficacy of immunotherapy. The faced challenges are the nonspecific modulation, which might have an unfavorable system cytotoxicity whereas specific methods may generate sub-therapeutic responses through work against some targets.^14^ The apparent dichotomy provides opportunities for metal particles platforms with unique properties to overcome the limitations of current treatments and boost the efficacious immune response to cancer.^15^ It can not only passively target tumors through a high osmotic retention effect, but also enhance active targeting by surface modification of targeted molecules.^16^

**Fig. 1:**
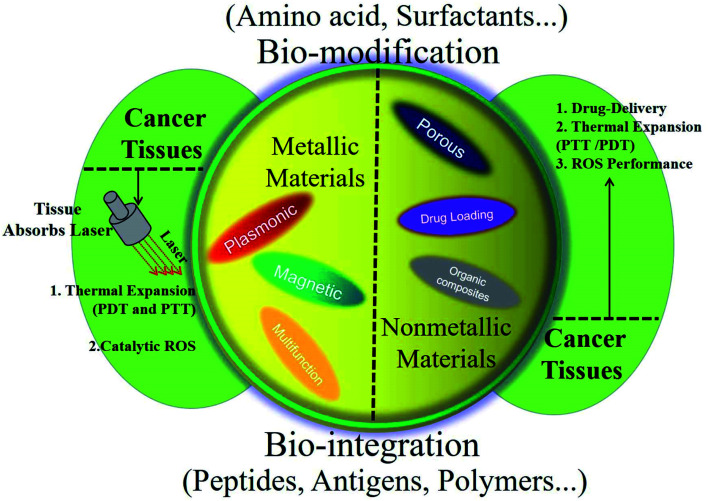
Schematic illustration of the utilization of metallic materials as efficient composites in cancer immunotherapy and their fabrication procedures.

As being entirely utilized in immunotherapies, the efficient delivery system requires a controllable approach while one or more applied drugs, antibodies, immunomodulators, or functional molecules to tumor sites (Fig. 1). After that, it could achieve enrichment, regulate local immunity, improve immunosuppressive microenvironment and the effect of tumor immunotherapies. The efficient metal-based functional materials can be roughly divided into pure-phased inorganic materials, organic materials, and efficient composites. Also, the nonmetallic materials can be functional composites in biological and biomedical applications in form of efficient agents in immunotherapies. Inorganic metallic materials could have clear chemical properties, controllable shape and size, easy to design, and unique optical characteristics. All of these specifics could match or integrate with target tissues leading to further advancement in cancer immunotherapies, vaccine development and auto immunotherapy. In this review, metallic materials are divided into pure metal particles, metal oxide, metal chalcogenides and metal-based heterostructures. The nonmetallic materials mainly refer to silicon-based and carbon-based nanomaterials. The organic materials mainly represent liposomes and polymer nanomaterials, which have the advantages of cell affinity, biocompatibility and low cytotoxicity. In this paper, we will mainly review the research progress of this type of nanomaterials in cancer immunotherapy.

## Pure-phased metal nanoparticles (NPs) as effective agents in cancer immunotherapies

Due to the obtained unique physical or chemical properties,^17,18^ pure-phased metal nanoparticles (NPs) can be engineered for use in many therapeutic applications including cancer immunotherapy.^8,19^ With a size range of 1 to 100 nm, a higher surface area to volume ratio and advantageous delivery kinetics, illustrated through a related mechanism, metal NPs exhibit positive attributes.^20–22^ So the designs of metal NPs could be customized to an intended application *via* modulation of particle properties, which include size, morphology, shape and surface charge (positive or negative).^23,24^ There have been early studies that focused on metal NP utilization in drug delivery to target tumors through the enhanced permeability and retention effect, in which, it could be further enhanced by conjugating tumor-target antibodies against metal NPs as much as possible.^25–27^ Therefore, the metal NPs are considered as one kind of substitutes with particularly advantages in cancer immunotherapy applications due to the precision with which their size, shape, charge, and surface modification can be controlled.^28^ At the same time, compared to nonmetallic nanoformulations of similar sizes, the higher density metal NPs are more readily taken up by cells due to the chemical bonding or physical interaction (surface functional groups), which could provide a benefit for cancer immunotherapy strategies.^29^ According to metal NPs' full characteristics, it has the potential of dense surface functionalization and unique optical properties, and can be used in optical or heating based therapy, for tumor ablation and immunotherapy mediated by metal NPs.^30,31^ In the real diagnostic treatments, cancer cells could recognize the relative matched antigens or molecules, in which, it would accord to the monitored expression of tumor-specific (mutant protein) or tumor-associated (up-regulated protein) antigens on tissue surface through biological interactions.^32,33^ In this process, metal NPs enhanced the immune response by improving antigen uptake by dendritic cells (and other antigen precursors), thereby improving the resulting anti-tumor cytotoxic T cell response.^8,34^ For instance, Au NPs are considered as one classical pure-phased metal NPs with controllable morphology and particle size, large specific surface area, biocompatibility, adjustable surface function with optical and electrical.^35^ It is used in biomedical fields, especially in tumor therapy and imaging applications.

**Fig. 2:**
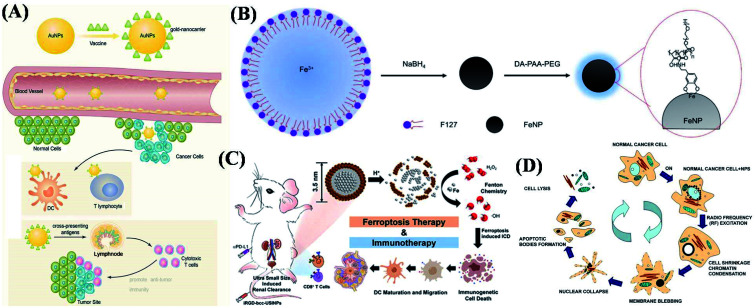
Metal-based nanomaterials as candidates for cancer immunotherapy. (A) Vaccines connect to and display on the surface of Au NPs to become a vaccine–Au NP complex; then, the complex penetrates blood vessels and delivers the vaccine targeting cancer cells to enhance the immune response. (B) Schematic illustration of the synthesis and surface modification of PEGylated Fe NPs. (C) Intravenous injection of iRGD-bcc-USINPs at three doses effectively suppresses tumor growth, and develops strong immune memory in immunotherapy. (D) Schematic diagram of HeLa cell with the C–Co-NPs apoptotic process under RF excitation. These are reprinted with permission from He *et al.* (2021, FRONTIER), Chao *et al.* (2019, ACS), Liang *et al.* (2021, ACS) and Xu *et al.* (2008, IOP), respectively.

Chen *et al.* reported that Au NPs have different influence while they were used in delivering antigens and observed significant serum antibody response procedures. The size and morphological factors are illustrated comprehensively.^36^ Similarly, Au NPs are used as a radio-sensitizer in tumor radiotherapy, in which, the size factor of Au NPs played one important role because it can enhance the radiation effect while using bigger ones. Combined with immunotherapy, this effect was one of the important issues in preliminary drug screening. In addition, Zhang *et al.* showed that modified Au NPs with different sizes (polyethylene glycol-PEG coated Au NPs) can significantly reduce the survival rate of cancer cells after γ-ray irradiation, and PEG-Au NPs (12.1 and 27.3 nm) have high radiosensitivity, which has important guiding significance for possible radiotherapy and drug delivery. According to immunotherapy treatment, lymph node localization is an important factor in cancer immunotherapy, because the morphology of lymph nodes is one of the key criteria for evaluating immune response. For further investigation, Sun *et al.* designed theragnostic glycol-chitosan-coated Au NPs (GC-AuNPs) as a photoacoustic contrast agent and tumor antigen delivery vehicle, which highlighted lymph nodes in ultrasound-guided photoacoustic (US/PA) imaging systems. Moreover, the ovalbumin epitope was conjugated with GC-Au NPs for delivering tumor antigen to lymph node resident macrophages. *In vitro* studies proved the vigorous endocytosis activity of J774A.1 macrophage and consequent strong photoacoustic signals from them. The macrophages also presented a tumor antigen when GC-Au NPs were used for cellular uptakes. As shown in Fig. 2A, it tested the cellular uptake of GC-Au NPs in macrophages. After the lingual injection of GC-Au NPs into healthy mice, cervical lymph nodes were visible in a US/PA imaging system with high contrast. Simultaneously, GC-Au NPs were able to deliver tumor antigens to cause macrophages to present the epitope at targeted lymph nodes, which would be valuable for cancer immunotherapy. Hwang *et al.* reported gold NPs (∼6 nm) that were pH-responsive and they were utilized as photothermal agents in biological applications. In which, the aggregates of particle units were responsible for the liquid pH environments. The coupled plasmonic mode of aggregates can be efficiently exploited for photothermal cancer therapy using a longer excitation wavelength.^37^ In another research, Lee *et al.* demonstrated that Au NPs and ferritin NPs could induce a cytotoxic T-lymphocyte (CTL) response against the model antigen when co-administered with CpG. This effect was abscopal in that the local treatment provided systemic immune protection and prevented RFP-expressing melanoma growth *in vivo*.^38^ Silvestrini *et al.* demonstrated that 15 nm CpG–Au NPs could be formulated with antigens and resulted in a substantial increase in IgG2a antibody titers, as well as improved T cell activation, leading to reduced tumor growth and improved survival in a lymphoma model system completely.^39^ The efficacy of combining metal NPs induced the ablation with chemotherapy and/or immunotherapy has been demonstrated using metallic NPs in preclinical studies.^40–43^ In one study, Au nanorods conjugated with Y-shaped CpG facilitated ablation and were co-delivered with doxorubicin. The therapy induced the production of IL-6 and TNF-a, resulting in a reduction in tumor volume *in vivo*. In a separate study, the application of CpG and doxorubicin (Dox) in combination with copper ion-mediated ultrasound was found to improve systemic anti-tumor immunity more than Dox alone.^44–46^ In addition, by using Cu–Dox–CpG composites as an efficient agent in mice treatments, it exhibited increased levels of leukocytes, CD^4+^ and CD^8+^ T cells, as well as decreased levels of immune-suppressive MDSCs.^47^ These copper-based particles were further tested in combination with ultrasound ablation, CpG, and PD-1, successfully.

In further works, the magneto-thermal therapy technique was used in cancer therapy *via* heating response under alternating magnetic field. It was an effective method for local tumor ablation, but it is not effective for metastatic tumors. Chao *et al.* have reported pure-phased Fe NPs with high magnetic saturation strength, which were modified by biocompatible polymers.^48^ NPs were stable in aqueous solutions and can be used as a super effective magnetic heating reagent to generate enough heat at low power as shown in Fig. 2B. In real works, the local or intravenous injection of Fe NPs was used to enhance the accumulation in the tumors. It was helpful for focusing on the tumor's constant magnetic field locally, in order to achieve effective MH ablation of the tumor. Due to the low production efficiency of reactive oxygen species (ROS), iron-based NPs need to be used in conjunction with other therapeutic methods or high-dose applications in order to achieve the purpose of effective treatment. Liang *et al.* synthesized ultrasmall single-crystal Fe NPs (bcc-USINPs) that stayed stable in a normal physiological environment but were highly active in a tumor microenvironment because of the selective acidic etching of a Fe_3_O_4_ shell and the exposure of the Fe (0) core.^49^ The bcc-USINPSs could efficiently induce tumor cell ferroptosis and immunogenetic cell death at a very low concentration. The process of immunotherapy is shown in Fig. 2C. Along with expanding investigations, the supplementary works in applying pure-phased metal NPs in cancer therapy. Xu *et al.* prepared cubic graphite carbon-coated ferromagnetic cobalt NPs (C–Co-NPs) with a diameter of about 7 nm by catalytic chemical vapor deposition (CVD). It can be thought of as one metal composite and considered as one merged-practical property (metallic and nonmetallic). These coated magnetic cobalt NPs showed higher specificity in radio frequency absorption and heating response. The schematic representation is presented in Fig. 2D, it shows the process used for targeting the cancerous cells, intracellular delivery of the C–Co-NPs and the inducement of apoptosis under RF excitation.^50^ Compared with the single property, structure and function of pure-phase metal NPs, metal-based metallic heterogeneous structures or composites are new advanced materials with unique charm, which can integrate two or more different functional nanomaterials into a whole, so as to obtain more excellent properties. The most common metal-based heterostructured NPs are made from precious metals, such as Au@Ag, Au@Pd, Au@Pt and so on. Their properties are also closely related to the structure and size of the NPs.

## Metal-based heterogeneous structures as functional candidate toward potential possibility in cancer therapies

**Fig. 3:**
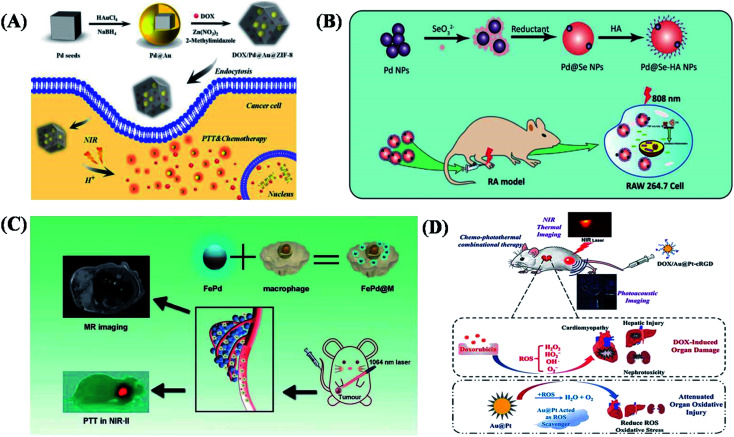
Metal-based heterogeneous structures as functional candidates towards potential possibility in cancer therapy. (A) Schematic illustration of the formation of DOX/Pd@Au@ZIF-8 and its application in pH- and NIR-triggered chemo-photothermal synergistic treatment of cancer cells. (B) The synthetic process of palladium (Pd) and hyaluronic acid (HA) integrated into selenium (Se) nanoparticles (Pd@Se-HA NPs) and therapy for rheumatoid arthritis (RA) by combination therapy inhibiting the macrophage inflammatory response *in vivo*. (C) Ultra-small iron–palladium (FePd) NPs with near-infrared-II (NIR-II) region photothermal response for targeted tumor photothermal therapy and magnetic resonance imaging. (D) Schematic illustration of chemo-photothermal combinational therapy for tumors using multifunctional Au@Pt NPs. These are reprinted with permission from Yang *et al.* (2017, RSC), Zheng *et al.* (2021, Chinese Pharmaceutical Association), Yang *et al.* (2019, RSC) and Yang *et al.* (2017, ACS), respectively.

Ascribed to metal characteristics in physical and chemical properties, metal-based heterogeneous NPs have attracted much attention because of their high photothermal conversion efficiency, special surface properties and mixed electronic transferring characterizations.^50*b*^ By compared with the traditional single metal NPs, the resulting bimetallic NPs have excellent physicochemical properties of the coated metal while maintaining the original metal core properties. With the cooperation of the two or more different metal compositions, they could exhibit more excellent special synergistic properties in reactions. They were distributed to the novel metal nanozyme and corresponding high catalytic performance in cancer imaging and therapy would significantly promote the generation of the new subdiscipline of nanomedicine by rationally integrating catalytic chemistry with clinical theranostic nanomedicine.^50*c*^ Yeh *et al.* reported Au NR-in-shell NPs synthesized by introducing silver into gold by displacement reaction.^51^ Compared with simple Au NRs, the resulting composites had higher photothermal conversion efficiency and poor nonlinear optical imaging in the near-infrared region. In addition, its near-infrared absorption range can be extended to 400 nm to 900 nm so that the heterogeneous bimetallic NPs can be used as photothermal reagents in tumor and immunotherapy.^52^ Wang *et al.* reported Au@Ag–Au NPs that were synthesized by a convenient, wash-free method with an adjustable shape and a spherical structure with an absorption spectrum that could be used for the photothermal therapy of tumors.^53^ As shown in Fig. 3A, Yang *et al.* reported metal-based efficient composite based on the metal–organic skeleton, which encapsulates Pd@Au NPs and doxorubicin (DOX) for pH and NIR-triggered synergistic chemotherapy–photothermal therapy together in cancer cells.^54^ As illustrated in fabrication, metal integrated with nonmetallic composite was also investigated and used in the relative application in further works. Such as Chen *et al.* combined palladium NPs with selenium NPs in order to obtain a composite system (Pd@Se–HA NPs), which could not only scavenge hydroxyl radicals (OH) but also provide a photothermal effect eventually. In which, Pd@Se–HA NPs were constructed by a simple self-assembly method as shown in Fig. 3B. Se NPs were electrostatically bonded to Pd NPs, and hyaluronic acid (HA) was connected to heterogeneous NPs through ester bonds to provide macrophage targeting ability. As one utilized approaches in cancer therapies, these kinds of physical properties could be merged with immunotherapy antigen or target proteins. As shown in Fig. 3C, Yang *et al.* reported ultra-small bimetallic FePd-loaded macrophages for targeted tumor photothermal therapy in NIR-II bio-windows and magnetic resonance imaging.^55^ In addition, the constructed porous Au@Pt structure was further explored for the properties of porous Au@Pt NPSs in relieving the oxidative stress damages as well as in tumor growth inhibition by chemo–photothermal co-therapy. The porous structure offered the possibility while using this structural space for medicine loading such as doxorubicin (DOX). This nanocarrier (DOX/Au@Pt-cRGD) showed controlled drug release behavior.^56^ As shown in Fig. 3D, the absorbance peak of Au@Pt structure in the near-infrared (NIR) portion provided the capacity for *in vivo* photoacoustic imaging and the high photo-conversion efficiency, which make Au@Pt a suitable carrier for photothermal therapy (PTT).

Through the above investigations, metal-based metallic NPs or composites already have been applied in different fields because of their multi-functional properties from different compositions. Through the atomic orders in heterogeneous metallic NPs, the possible synthesis approaches could be according to metal ions' reaction potential under a reasonable reducer. The improved capacities were mainly determined by inherent factors such as size distribution, and reactivity, which enhanced their clinical and therapeutic applicability. Besides pure metal or heterogeneous metallic materials, metal oxides and metal chalcogenides also have long-lasting immune effects on cancer cells *in vivo* models. In which, the multiple effects of metal oxide NPs on macrophages, which are dependent on the type of metal and the route of synthesis. Although to a large extent, metal oxide NPs are considered to be drug transporters, it still has the potential of immunotherapy because they can evoke the pro-inflammatory or anti-inflammatory effects of macrophages. It may be much more necessary for macrophage morphogenesis in cancer, wound healing, infection applications and autoimmunity.

## Metal oxides materials and chalcogenides as functional composites in full utilization toward cancer immunotherapies

**Fig. 4:**
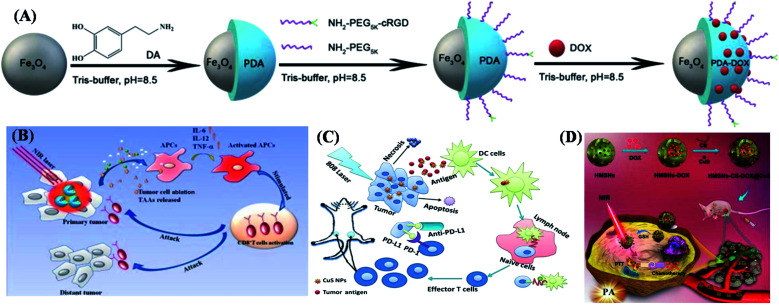
Metal oxide materials and chalcogenides as functional composites towards cancer immunotherapy. (A) Schematic illustration of the synthesis of DOX-loaded Fe_3_O_4_@PDA–PEG–cRGD composite particles. (B) CuS@OVA–PLGA–NPs presented as a promising strategy for metastatic tumor therapy. (C) Polyethylene glycol-modified CuS NPs (CuS NPs–PEG–Mal) with stronger antigen adsorption capacity; the therapeutic strategies provide a simple and effective treatment option for metastatic and recurrent tumors. (D) Schematic illustration of the concept behind using HMSNs–CS–DOX@CuS for thermal-PA imaging-guided tumor chemo-PTT therapy. These are reprinted with permission from Fan *et al.* (2019, Dove Press), Chen *et al.* (2020, Elsevier), Wang *et al.* (2019, ACS) and Niu *et al.* (2021, Elsevier), respectively.

During current investigations, immunotherapies not only could combine with cancer therapy approaches, but also be immersed with biological treatments in further applications. According to the inherent properties of metal oxides and metal chalcogenides, there are some physical functions (NIR, catalytic response, and structural distinguish) that were utilized for fabricating extended applications eventually. Here, PTT is an infrared-mediated approach that affects normal tissue cells and the immune system and is also a promising approach. It mainly has the advantages of high targeting, high efficiency, minimally invasive and few side effects, being used in the treatment of various types of cancer.^57^ However, the use of PTT alone has limitations: light penetration depth is limited, it resulted in incomplete elimination of cancer cells, and the residual edge of the treatment area may lead to the recurrence of the disease.^58,59^ Immunotherapy plays an important role in the treatment of metastatic tumors. The combination of physical effect and influence can not only eliminate tumor cells through hyperthermia, but also improve the release of antigens in the body to enhance the immune response. Presently, a variety of metal oxide NPs has been successfully prepared in combination with PTT in order to treat cancer immunity. Also, superparamagnetic Fe_3_O_4_ was used in catalytic effect and metal ions inter transfer due to its unique magnetic response and different metal valent-states,^60,61^ can be used in PTT and Magnetic Resonance Imaging (MRI) as a tool for tumor diagnosis and treatment. Iron oxide NPSs have been developed as MRI contrast agents and are healthy for humans. In addition, the proper surface functionalization was used to prevent them from being cleared out of circulation by the immune system. The study successfully prepared multifunctional Fe_3_O_4_@PDA–PEG–cRGD–DOX NPSs that combines MRI diagnosis with PTT and chemotherapy. As shown in Fig. 4A, the schematic illustration of the synthesis of metal oxide-based functionalized composites (DOX-loaded Fe_3_O_4_@PDA–PEG–cRGD) was exhibited in the preparation step.^62^ The results showed that the nanosystem had biocompatibility and excellent photothermal conversion ability. *In vivo* experiments showed that the multifunctional NPs had good MRI contrast and could effectively target tumor tissue. In which, it had good EPR and active targeting effect, and could significantly inhibit the growth in the reproduction of tumor tissues. Therefore, these composites can be used as a potentially effective vector for tumor diagnosis and combined chemotherapy or PTT.^63,64^

Copper-based chalcogenides (CuS NPs) were considered as one effective composited in NIR window material. It can be used not only as a photothermal medium of tumor hyperthermia but also as an antigen trapping agent to induce an anti-tumor immune response. CuS NPs–PEG–MAL was used in combination with immune checkpoint blockers (Fig. 4B). The results showed that CuS composites hyperthermia could significantly increase the level of inflammatory cytokines in serum, leading to the formation of tumor immune microenvironment. It could mediate PTT while increasing the number of tumor-infiltrating CD8+T cells. It inhibited the growth of primary and distant tumor sites in the 4T1 tumor model under the action of co-anti-PD-L1. This approach provided a simple and effective treatment option for both metastatic and recurrent tumors.^65^ Polylactic acid glycolic acid (PLGA) NPSs with different surface chemical structures have been reported to enhance the antitumor efficacy of immunotherapy by capturing tumor-derived protein antigens (Tdpa) released in the tumor environment after radiotherapy and then delivering them to antigen-presenting cells (Apc) to enhance the antitumor efficacy of immunotherapy.^66,67^ In the study, however, PLGA NPs have only one function, antigen capture, and must be combined with additional radiation therapy to improve the efficacy of immunotherapy.^68,69^ CuS NPs with surface functionalized modifications have two functions: (1) as photothermal coupling agents for tumor ablation;^70^ (2) as an antigen trapping agent, the antigen released during the heating process is absorbed and transported to dendritic cells (DC). Combined with an immune checkpoint inhibitor (anti-PD-L1), the efficacy of surface-functionalized CuS NPs was evaluated under mediated hyperthermia to enhance tumor immunotherapy as shown in Fig. 4C. Combining chemotherapy and PTT into a single agent is an effective strategy to improve cancer treatment.^71^ From traditional approaches, hollow silica NPs due to their hollow characteristic could be used as the base carrier material to load the anticancer drugs, and the surface functionalization of chitosan (CS) and CuS NPs was performed to obtain HMSNs–CS–DOX@CuS (Fig. 4D). In this research, the role of CuS is introduced to prevent the sudden release of drugs into systemic circulations.^72^ This targeted delivery was achieved by CuS NPs as gatekeepers attached to the surface of hollow silica. When exposed to near-infrared laser irradiation, the metal composites generated energy that causes the S–S bond to break and accelerates drug release. In addition, these photothermal properties of composites make them suitable for PTT. In this work, chitosan (CS) was modified by mercaptoacetic acid to CS–SH, which is then covalently bonded with HMSNs *via* disulfide bonds. CS is a material suitable for drug delivery systems: widely available, low-cost, biodegradable, biocompatible and pH sensitive.^73,74^ Therefore, CS can be used to improve the biocompatibility and solubility of materials. Here, the anticancer drug was encapsulated on CS modified hollow structures with unreacted sulfhydryl groups bound to CuS *via* disulfide bonds. Disulfide bonds were known to exhibit reductive behavior and can be cleaved in the presence of reducing agents such as glutathione.^75^ The level of GSH in cancer cells is several times higher than in normal cells, so once the metal-based efficient composites reached the tumor, and the disulfide bonds connecting CuS might break completely. CuS mediated PTT heating will accelerate this effect if NIR radiation is applied simultaneously. This causes DOX release and induces apoptosis of cancer cells. CuS's strong NIR absorption further allows for thermal and PA imaging, thus enabling the system to have the therapeutic potential.^76^ A biocompatible titanium dioxide coated with a series of organic coatings has been reported. Ligand exchange reactions can be carried out on the outer encapsulated groups without interfering with the overall size or structural morphology of NPs. These NPs can be easily dispersed in water and can also be used to carry cytotoxic radionuclides for radioimmunotherapy, in which ultra-small NPs are essential for rapid kidney clearance. Titanium dioxide NPs are also effective as photosensitizers in tumor photodynamic therapy. After UV irradiation, titanium dioxide NPs can produce reactive oxygen species in tumor cells, and then attack cancer cells, thereby inducing cell apoptosis and producing anti-cancer activity. It can also be used as an alternative photosensitizer for PDT in tumor targeted therapy.^77,78^ Based on metal oxide NPs combined with the PTT approach has achieved some success in cancer immunotherapy response, the therapeutic performance of these mono-stimulatory drug delivery systems was affected due to low tumor specificity or insufficient activation of anti-tumor immune response. In order to achieve tumor-specific drug delivery, biological NPs were designed for drug delivery. They could be served as carriers for cancer drugs, as well as delivering proteins and immunotherapy components. Some of them have even been used as intermediaries for PTT/PDT directly destroying cancer cells without harming normal tissue cells.

## Nonmetallic materials as alternative composites exhibit capacities in cancer treatment as immunotherapies

**Fig. 5:**
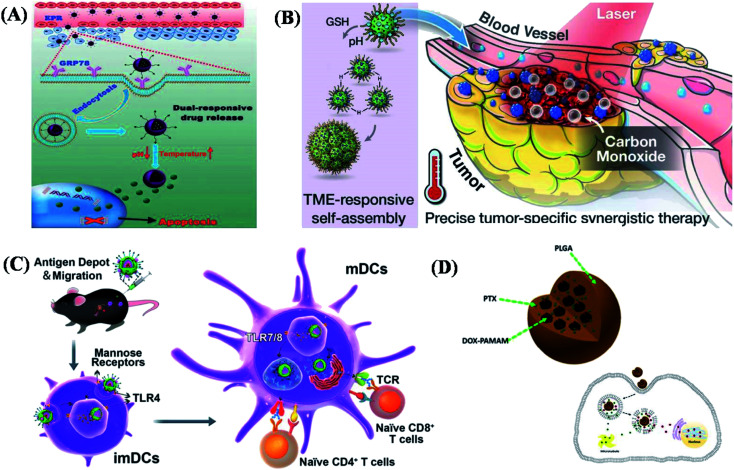
Using nonmetallic composites as functional agents in cancer therapy and further immunotherapy. (A) Schematic illustration of the smart NPs with prolonged blood circulation, enhanced tumor accumulation, efficient cancer cell uptake, pH- and temperature-responsive release of PTX, and the capability of targeting breast cancer cells. (B) Schematic illustration of the Mn_2_(CO)10-loaded and POM surface-modified hollow mesoporous organosilica nanoplatform, HMOPM-CO, for tumor microenvironment (TME)-responsive self-assembly and precise synergistic therapy. (C) Illustration of targeted co-delivery of antigen and agonists by PCL–PEG–PCL hybrid nanoparticles for cancer immunotherapy. (D). Schematic illustration of the structure of the nano-in-nano polymer–dendrimer nanoparticle-based drug delivery nanosystem, the cellular uptake of NPs, and the controlled drug release from the nanosystem. These are reprinted with permission from Niu *et al.* (2018, Taylor & Francis), Tang *et al.* (2018, ACS), Zhuang *et al.* (2019, ACS) and Zhao *et al.* (2017, ACS), respectively.

At present, tumor immunotherapy is considered as the effective tumor treatment strategy as much as possible. Fortunately, the immune system is not responsive to tumor antigen stimulation as one of the obstacles to clinical application in tumor immunotherapy. Here, the metal-based functional materials have already been studied and utilized in immunotherapy due to their irreplaceable advantages of carrying antigens to specific sites and stimulating immune responses. As shown in Fig. 5A, the development of both the safety and effect of cancer vaccine agents is a common focus in the field of cancer immunotherapy.^79^ By using semiconductors, among many fluorescent materials such as carbon dots (CD) possesses imaging capacity. Photoluminescent CDs could combine with tumor protein antigen model ovalbumin (OVA) as a vaccine adjuvant to emit red, yellow and green light at different excitation wavelengths. These CDs can actively promote antigen uptake and effectively promote the maturation of dendritic cells (DCS) as shown in Fig. 5B. CDs–OVA composites can effectively enhance the expression of DC costimulatory molecules CD80 and CD86, and promote the production of tumor necrosis factors. Furthermore, this CDs–OVA vaccine can be effectively endocytosed and processed by *in vivo* immune cells to induce a strong antigen-specific cellular immune response. CDs exhibited highly uniformed size distribution with surface passivation using PEG and surface functional groups have been synthesized. It proved that the CD surface was successfully bound to model tumor antigen OVA (Fig. 5C). These nonmetallic composites could induce DC maturation and secretion of related cytokines. At the same time, CDs can not only prevent antigen inactivation in a complex physiological environment but also help the antigen to be most likely to be endocytosis. Therefore, nonmetallic materials (CDs–OVA) can significantly inhibit the growth of established OVA-expressing B16 melanoma.

It is an effective strategy for cancer treatment through the combined administration of multiple chemotherapeutic agents with different mechanisms of action. Additionally, a polymer–dendrimer hybrid in form of NPs was developed for efficient and controlled co-delivery of two models (chemotherapy drugs, doxorubicin and paclitaxel) NP-based nanosystems have been developed for efficient and controlled co-delivery of different models as shown in Fig. 5D. The results of *in vitro* cytotoxicity showed that dual-drug loaded nanosystem had a better antitumor effect than single-drug loaded nanosystem or free dual-drug loaded combination by optimizing the drug ratio.^80^ In addition, carbon nanotubes (CNTs) have unique structures and properties, including high crisscrossing, large specific surface area, rich surface chemical function and dimensional stability at the nanoscale. They can be used as vectors and mediators for cancer therapy, as well as protein delivery and immunotherapy components. It can be used as intermediaries for PTT and PDT to directly destroy cancer cells without damaging normal tissue cells.^81–83^ Fadel *et al.* proposed a carbon nanotube-polymer composite that can effectively amplify T cells as artificial antigen-presenting cells. The antigen was attached to a bundle of carbon nanotubes and the CNT complex was bound to polymer NPs containing magnetite and T-cell growth factor.^84^ Fan *et al.* investigated systemic antitumor responses to intracerebral injection of CpG and CNTs. They demonstrated that intracranial CNT–CPG therapy inhibited not only the growth of brain tumors but also subcutaneous melanoma.^85^ Based on these results, it suggested that intracerebral CNT–CPG therapy can be used to treat not only gliomas but also metastatic brain tumors. Villa *et al.* investigated the possibility of using CNTs as antigenic carriers to enhance the polypeptide immune response.^86–90^ Meng *et al.* connected multi-walled carbon nanotubes to tumor lysis proteins in order to improve the effect of immunotherapy using tumor cell vaccines. In a mouse model, the tumor therapeutic effect and cell anti-tumor immune response of tumor, lysine-labeled CNTs were significantly improved. These treatments have been shown to be effective in inducing, enhancing, or suppressing immune responses.^91^ The successfully designed systems with mixed metal NPs were effective for efficient co-delivery of multiple chemotherapeutic drugs. The antitumor efficacy of the dual drug delivery system was evaluated *in vitro* in different cancer cells.

Although some nanocarriers have been reported to combine the delivery of different anticancer drugs, there are still some problems to be solved. In order to deliver drugs efficiently and minimize the side effects caused by the delivery vector itself, the drug delivery rate is required to be high enough. Since the release kinetics of different drugs may greatly affect the therapeutic effect of drug combinations, the drug delivery system is also needed to achieve controlled release.

## Pure-phased organic composites as functional agents in cancer immunotherapies

As a critical organic component, dendritic cells (DC) were currently used as cell vaccines in clinical trials of tumor immunotherapy.^88–91^ It reflected the recognition of the critical role of DC in initiating anti-tumor immunity, which led to the development of several strategies to target vaccine antigens to DC and triggered anti-tumor T cell responses. The encapsulation rate can be increased by increasing the antigen concentration of tumor lysate. However, increasing the antigen concentration reduces the encapsulation efficiency. In addition, NPs with higher initial protein content resulted in a greater cumulative release. Poly-*co*-glycolic acid (PLGA) NPs are attractive vectors of protein antigen delivery, which can effectively induce stimulation and maturation of DC,^92^ which can not only enhance antigen processing and immunogenicity or improve antigen stability, but also target delivery and slow release of antigens.^91^ Mature stimulated DCs showed a strong enhancement in stimulating the original autologous T helper cells and secreting cytokine numbers.^92,93^ At the same time, fucoidan was a sulfurated polysaccharide purified form, which has a variety of immunomodulatory effects, including promoting antigen uptake and enhancing antiviral and anti-tumor effects. It was an excellent adjuvant to induce Th1 immune response and CTL activation, which may contribute to the development of tumor vaccines.^94,95^ A laser/glutathione (GSH) activated tumor-penetrating system was reported for efficient immunotherapy. The system has a variety of therapeutic approaches that enable efficient tumor immunotherapy, combining reversal and enhancement of tumor immunogenicity. In which, the core of the system was synthesized by the coordination of zinc ion with glutathione-activated oxaliplatin prodrug and carboxyl phthalocyanine. Here, coordination polymer NPs were fabricated and coated with phospholipid bilayer.^95^ Similarly, in order to overcome the challenges of cellular immunotherapy, liposomes have been well developed that can combine with tumor-derived RNA to produce personalized tumor RNA NPs with considerable amplification capacity. In this fabrication, the combination therapy with systemic immunotherapy RNA-NPs might extend its therapeutic potency although immunotherapy was only effective in a small percentage of cancer treatment.^96^ RNA-NPs also induced up-regulation of activation markers on tumor antigen precursor cells and effective proliferation of antigen-specific T cells.

## Conclusion

We presented a detailed overview of the role of metallic materials as one of the efficient composites for cancer therapies. Especially, during the utilization of metal-based functional materials, the metal elements could mix with each other during synthesis and alloying processes. We presented and summarized previous investigations that referred to cancer immunotherapy through using metallic materials and their related structural fabrication techniques. Combining immunomodulators with NPs for cancer immunotherapy is a very potential clinical therapy, but different immunomodulators require nanocarriers to have different loading efficiency and the universal type. As mentioned, new conceptions for biological applications and further investigation for cancer therapies, metal-based functional materials should play a key role in the innovations about biological or medical applications. Distinguished with pure-phased metallic materials itself, the particularity of heterogeneous metallic materials can be treated as photo-sensitizers, thermal-sensitizer, and drug loading agents, which could fix the whole system as one flexible substrate. This should be the preferred option in integration towards cancer cells. This would not only prevent sedimentation but promote candidates' recycling and also alleviate the cost of reduced mass transfer between the target cancer issues and reactants. In the long run, it is reliable for the collective optimization of metal-based sensors, reaction media and reactors, and their practical applications for cancer therapy valorization. In spite of the promised future research field, the developed multifunctional metallic materials and their systems for cancer therapy applications are limited.

The research progress of cancer immunotherapy can guide immunotherapy, which will contribute to the innovative treatment of tumors. Blocking the way that tumor cells try to escape immune surveillance is the key to the success of immunotherapy. Metal or nonmetallic materials, even metal oxides, have achieved success in a variety of immunotherapy applications, from delivering immunomodulatory materials to inducing the release of tumor antigens after local ablation. Therefore, in order to deeply explore the interaction between metallic materials and immunotherapy systems, the transport mechanism and targeting tissue diagnostic procedures in tumor cells should be improved and optimized for *in vivo* biological applications. It could break through the key technology of tumor immunotherapy, which is the key research direction of nano drugs in tumor immunotherapies. They might be able to enhance the magnitude of the immune response induced using metal-based immune approaches. In any case, there is still a lot of room for progress in cancer immune responses with NPs based on various materials.

## Conflicts of interest

The authors declare no conflict of interest.

## Supplementary Material
